# Design of digital economy consumer psychology prediction model based on canopy clustering algorithm

**DOI:** 10.3389/fpsyg.2022.939283

**Published:** 2022-08-02

**Authors:** Yue Zhang, Peng Ruan, Jingfeng Zhao

**Affiliations:** ^1^School of Economics and Management Northwest University, Northwest University, Xi’an, China; ^2^The First Affiliated Hospital of Chengdu Medical College, Chengdu Medical College, Chengdu, China

**Keywords:** Canopy clustering algorithm, digital economy, consumer psychology forecast, predictive model design, digital consumption

## Abstract

With the continuous improvement of the level of science and technology, the popularization of the Internet and the development of applications, online consumption has become a major force in personal consumption. As a result, digital consumption is born, and digital consumption is not only reflected in transaction consumption at the monetary level. Like some intangible services similar to the use of dating software, it can also become digital consumption. In this environment, a new economic concept, the digital economy, has emerged as the times require. The digital economy helps to achieve the rapid optimal allocation and regeneration of resources and achieve high-quality economic development. Therefore, as a new economic form, the digital economy has penetrated into all fields of human society. The Canopy algorithm is a fast clustering technique that requires only one pass through the data technology to get the results. But it is inaccurate for large-scale data clustering. Therefore, when analyzing the data, it is necessary to use the Canopy algorithm for preliminary clustering, and then combine with other algorithms or model software for refinement. This article introduces the development of the digital economy in the Internet era. It conducts a theoretical discussion on consumer psychology in the context of the digital economy. It introduces the basic calculation formula of the clustering algorithm and the algorithm flow of the Canopy clustering algorithm. It does model optimization for the Canopy clustering algorithm. On this basis, it designs questionnaires for experimental design. The indicators are divided into commodity attributes and consumer psychology. It builds a consumer psychology prediction model and tests the prediction results. The results show that the maximum difference between the prediction results of digital economy consumption psychology based on the Canopy clustering algorithm and the actual results is 0.047. It can be shown that the psychological prediction model of digital economy consumption based on the Canopy clustering algorithm has certain practicability.

## Introduction

### Background

As the main economic form after the agricultural economy and the industrial economy, the digital economy has caused fundamental changes in the global economic environment and economic model due to its fast development speed, wide radiation range and deep influence. It has become a key force to reorganize global factor resources, reshape the global economic structure, and change the global competition pattern. According to data from the China Academy of Information and Communications Technology, the scale of China’s digital economy reached 27.2 trillion yuan in 2017, ranking second in the world. Its average annual compound growth rate reached 38%, far exceeding China’s GDP growth rate over the same period. As one of the fundamental drivers of economic development, household consumption is also affected by Internet big data. That is to say, residents’ consumption concepts, consumption psychology, and their derived consumption behaviors are all influenced by technology industries such as Internet big data, artificial intelligence, and cloud computing. In this context, research on consumer psychology will help promote residents’ consumption and be beneficial to economic development.

### Significance

The digital economy has the characteristics of globalization. At present, the global economic development is in a weak state, and the Chinese economy is not optimistic. Overcapacity, excessive inventory, and increased risk of financial leverage have all led to increased downward pressure on China’s macro economy. Therefore, driving residents’ consumption has become a feasible path for stable economic development. Compared with the traditional statistical analysis, the Canopy clustering algorithm is faster and the data discrimination is more obvious. Based on the Canopy clustering algorithm, the digital economy consumption psychology prediction model is constructed to provide experimental data reference for the study of residents’ consumption psychology, which has certain theoretical significance.

### Innovation

(1) It uses the Canopy clustering algorithm to build a consumer psychology prediction model in the digital economy. It uses new data algorithms to study consumer psychology. It is a major innovation in statistical analysis of such problems. (2) Based on the Canopy clustering algorithm, it optimizes the model of the algorithm used in the experiment. Data analysis results are more practical.

## Related work

As a kind of economic form of globalization, digital economy has corresponding theoretical studies in various countries. Simon, investigated the development of the digital economy in different countries by measuring the market capitalization of selected countries using financial databases. The results showed that the companies with the largest market capitalization currently originate from the United States (Mueller et al., 2017). From the perspective of network information security, Teoh and Mahmood (2017) studied the security issues such as cyber threats and cyber risks brought by malware, leakage of personal information and organized cybercrime in the context of the prosperity and development of the digital economy. He believed that countries need to do a good job of national cybersecurity strategies. Markova et al. (2021) studied the economics and laws of corporate innovation activities in the context of the digital economy. He believed that the strategic goal of ensuring information security was to protect the vital interests of individuals and society from internal and external threats (Markova et al., 2021). Kazakov and Valijonov (2021) studied product competitiveness in the context of the digital economy. He believed that improving product quality management is a necessary condition to ensure competitiveness (Kazakov and Valijonov, 2021). The digitalization of products brings about the digital economy. In other words, in the digital economy model, products are also the key to competitiveness. Saxena et al. (2021) believed that consumer psychology and culture are closely related to consumer behavior. Therefore, it is very important to study the consumer’s consumption psychology for the design and positioning of the brand. From the perspective of consumer psychology, Ali (2019) believed that marketers should study the art of influencing the subconscious, so that consumers can participate in the cognition of products. Yao et al. (2018) used factor analysis and structural equations to test the mediation effect. Through the mediating effect of consumer psychology, he empirically analyzed the influence of clothing marketing experience environment on consumer intention and behavior. The results showed that the introduction image and service image have a positive impact on consumers’ intentions and behaviors. Shaw and Bagozzi (2017) believed that consumer researchers can better study consumer psychological processes through neuroscience techniques. In order to make the obtained data more practical, it is particularly important to choose an appropriate data analysis method. As a tool of data mining, cluster analysis has strong data processing ability. Lin and Xu (2020) proposed a road network division method based on Canopy-Kmeans clustering algorithm. In addition, he constructed a vehicle networking simulation platform based on Vissim simulation software with the real-time acquisition of the center latitude and longitude, the average speed and average density of the road sections (Lin and Xu, 2020). Zhu et al. (2021) used the k-means clustering segmentation algorithm to identify soybean canopy images from the original 2D images. However, the above studies are all descriptions of the digital economy and do not highlight the theme of the article. In addition, they have no deeper research on the design of consumer psychology.

## Brief introduction of digital economy and canopy-k-means clustering algorithm

### Overview of the digital economy and consumer psychology under the digital economy

**FIGURE 1:**
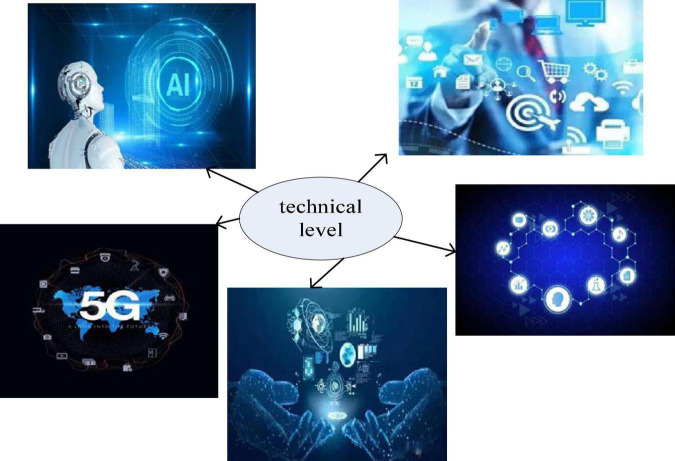
Areas covered at the technical level of the digital economy.

The digital economy refers to the identification-selection-filtering-storage-use of human beings through digital knowledge and information, guiding and realizing the rapid optimal allocation and regeneration of resources. This achieves the economic form of high-quality economic development (Alizadeh et al., 2017; Markova et al., 2021). From a technical perspective, the digital economy includes emerging technologies such as big data, cloud computing, Internet of Things, blockchain, artificial intelligence, and 5G communications (Rrustemi and Tuchschmid, 2020), as shown in Figure 1.

**FIGURE 2:**
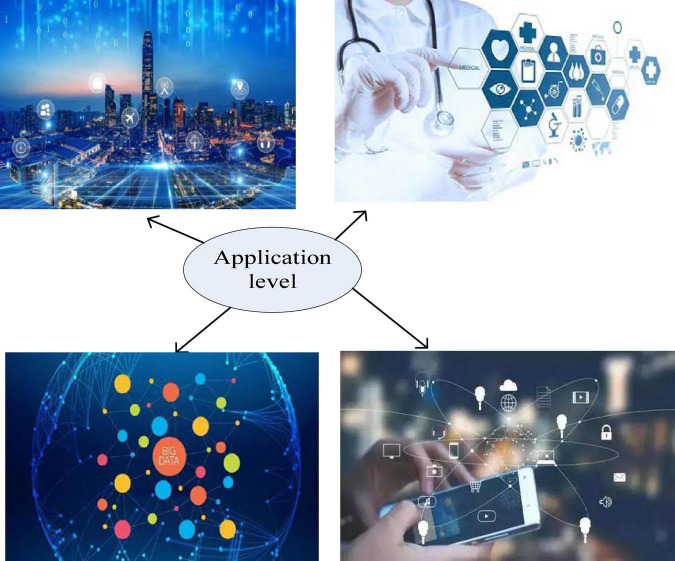
The areas covered by the application level of the digital economy.

As can be seen from Figure 1, the connotation of the digital economy is relatively broad. Any economic form that directly or indirectly uses data to guide resources to play a role and promote the development of productivity can be included in the category of digital economy (Wang and Shi, 2021). From the application level, the digital economy includes new retail, new manufacturing, medical and health data, finance, and smart cities, as shown in Figure 2.

Figure 2 shows the scope of the digital economy at the application level. It reflects that the digital economy has accelerated the penetration of digital technology, goods and services into traditional industries in multiple directions, multiple levels and multiple chains through continuously upgraded network infrastructure and smart devices and other tools. This greatly reduces social transaction costs. The digital economy has created a win-win environment for businesses and consumers because of its sound business model. New economic models also bring new consumption patterns. Compared with the traditional industrial era, consumption in the digital economy era pays more attention to the characteristics of crowd networks. Table 1 shows the consumption characteristics under different economic models.

**TABLE 1:** Consumption characteristics under different economic models.

	Industrial economy	Digital economy
Demand	Functional consumption	Data consumption
Frequency	One-time consumption	Continuous consumption
Scale	Single product consumption	Internet-connected consumption
Form	Individual consumption	Community consumption

**FIGURE 3:**
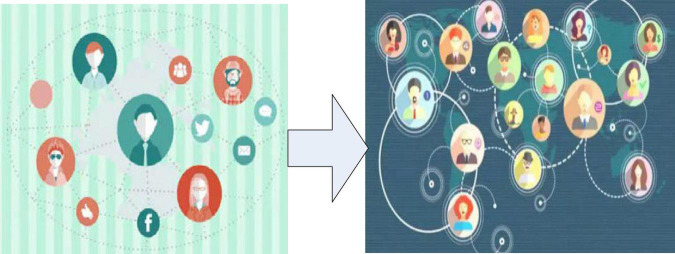
Crowd social network structure.

Table 1 shows the consumption patterns in the industrial age and the digital age. It can be seen that in the era of digital economy, people’s consumption characteristics are mainly concentrated on the Internet. The traditional consumption model chooses a single product more. Due to the analysis of big data, the consumption pattern in the digital age will lead to the consumption of related products. In addition, consumer demand has also changed from functional demand to data demand, and the consumption form has changed from individual consumption to community consumption. According to the effect of social network, when the network is large enough, information is connected to everyone on the network, individuals have their own social network, and there is a cross between networks. This resulted in today’s social network-like structure, as shown in Figure 3.

**FIGURE 4:**
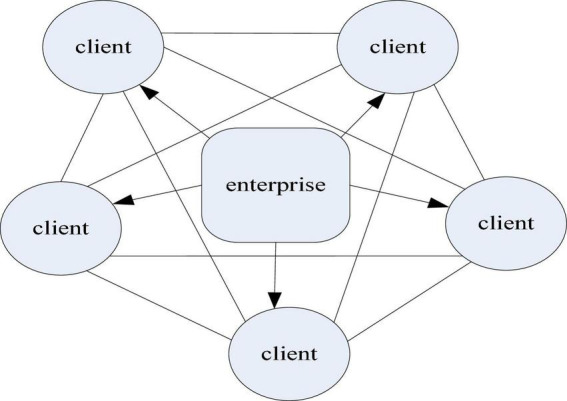
Enterprise customer link resources.

As can be seen from the structure diagram of the crowd social network in Figure 3, with the expansion of the aggregation scale of the crowd network in the social network, the information exchange becomes more extensive. Extensive information exchange on the one hand increases people’s awareness of the product, and on the other hand has a positive effect on the dissemination of the product. This has also become a fundamental condition for the development of the digital economy. In this context, it also drives the increase of enterprise customer link resources, as shown in Figure 4.

**FIGURE 5:**
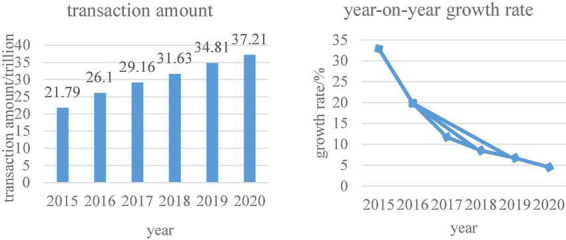
Trends in China’s e-commerce transaction volume.

Figure 4 shows the form of link resources for enterprise customers in the context of the digital economy. As can be seen from the figure, the information network between enterprise customers and customers can help enterprises to increase resource links. Therefore, enterprises under the digital economy model need to consider the integration of customer resources (Shelepov, 2020). It can also be seen that it is the development of the Internet that drives the development of e-commerce, thus giving birth to China’s digital economy model. According to statistics from China Business Industry Research Institute, the trend of China’s e-commerce transaction volume is shown in Figure 5.

**FIGURE 6:**
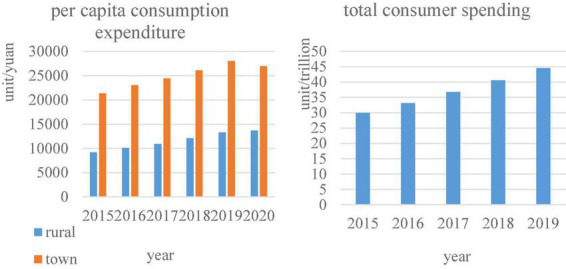
Per capita consumption expenditure vs. total consumption expenditure.

As can be seen from Figure 5, the total amount of e-commerce transactions is increasing year by year. Figure 6 shows data on per capita consumption expenditures in rural China, urban consumption expenditures, and total household consumption expenditures. This can also show consumers’ consumption preferences in the digital economy.

**FIGURE 7:**
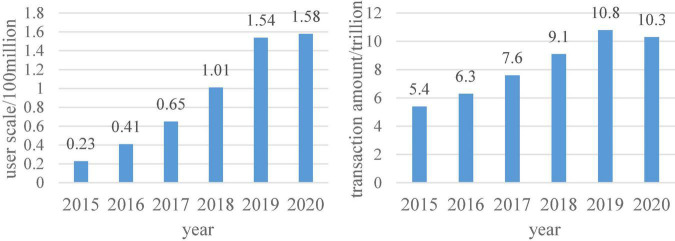
The scale of China’s overseas online shopping users and the scale of cross-border e-commerce transactions.

Consumer psychology is the mental activity of consumers when they look for, choose, buy, use, evaluate and dispose of products and services related to themselves. This mainly includes the consumer psychology of conforming, seeking differences, comparing, and seeking truth. With the development of the Internet and the expansion of the crowd network, the simplicity and convenience of digital consumption are favored by consumers. Under the operation of big data, the website will recommend personal preference content. They spend less time picking their personal favorites. This is one of the reasons for the popularity of digital consumption. According to relevant data reports, the scale of China’s overseas online shopping users and the scale of cross-border e-commerce transactions are shown in Figure 7.

According to the data in Figure 7, it can be seen that the number of overseas online shopping users has been increasing in recent years, and cross-border e-commerce will also grow steadily before 2020. This is due to the accelerated pace of life, which makes people’s lifestyle choices tend to be simpler and faster. Therefore, in this environment, digital consumption in the digital economy can better satisfy consumers’ consumption psychology.

**FIGURE 8:**
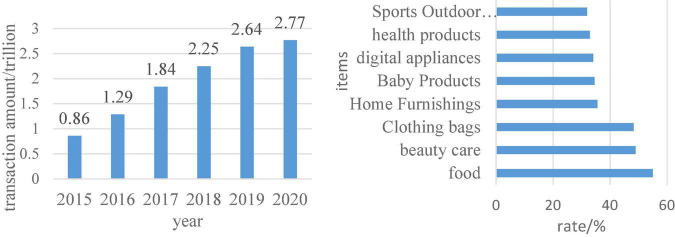
The scale of China’s imported cross-border e-commerce and the main products purchased by imported cross-border e-commerce in 2018.

Figure 8 shows the scale of China’s imported cross-border e-commerce and the market share of the main categories purchased by imported cross-border e-commerce in 2018. It can be seen from the data in the figure that the transaction volume of the import cross-border e-commerce market is gradually increasing. Food, beauty and personal care, clothing and bags were the most frequently purchased products by users in 2018. Judging from these two types of data, consumers’ consumption needs are mainly in terms of diet and personal care, and their pursuit of products is also more international. This is also a consumption advantage in the digital economy era.

### Clustering algorithm analysis

Clustering algorithm is a statistical analysis method to study classification problems. The principle of data analysis is to divide a large amount of data according to certain rules, and use the data to drive the required classes (Yuvaraj and Manimozhi, 2018). The main types of clustering algorithms include partition method, hierarchical method, density algorithm, graph clustering method, grid algorithm, and model algorithm. The main categories are shown in Table 2.

**TABLE 2:** Classification of clustering algorithms.

	Theory	Representative algorithm
Partitioning methods	Distance based	K-MEANS algorithm, K-MEDOIDS algorithm, CLARANS algorithm
Hierarchical methods	Perform a hierarchical decomposition of a given dataset	BIRCH algorithm, CURE algorithm, CHAMELEON algorithm, etc.
Density-based methods	Density based	DBSCAN algorithm, OPTICS algorithm, DENCLUE algorithm, etc.
Grid-based methods	Target a single unit	STING algorithm, CLIQUE algorithm, WAVE-CLUSTER algorithm
Graph theory clustering	Using the local connection features of sample data as the main information source for clustering	
Model-based methods	Each cluster assumes a model	

Table 3 shows the requirements and reasons for the clustering algorithm for real data. From the information summarized in the table, it can be concluded that the data in reality is often multi-dimensional, multi-angle and multi-conditional. The choice of clustering algorithm is often related to the characteristics of the data. Therefore, the use of clustering algorithms is often used to analyze data in the form of combinations (Khan, 2017; Kumar and Kumar, 2017).

**TABLE 3:** Clustering requirements and reasons.

Clustering requirements	
**Requirements**	**Cause**
Scalability	A large database may contain millions of objects
Different properties	Clustering of other types of data may be required, such as binary, categorical/nominal, ordinal, or a mixture of these data types.
Any shape	A cluster may be of any shape
Field minimization	Clustering results are very sensitive to input parameters
Dealing with “Noise”	Most real-world databases contain outliers, missing, or erroneous data
Record order	Some clustering algorithms are sensitive to the order of the input data
High dimensionality	A database or data warehouse may contain several dimensions or attributes
Constraint based	Real-world applications may require clustering under various constraints
Interpretability—usability	Users expect clustering results to be interpretable, understandable, and usable

Common clustering algorithms use data matrix, dissimilarity matrix and similarity matrix. The metric in the clustering algorithm is similarity. For different types of data, the similarity metrics it uses are also different. For some linear data, such as temperature, time, etc., it uses interval-scaled variables. Many clustering algorithms determine clusters based on Euclidean or Manhattan distance metrics (Bilozubenko et al., 2020). It assumes that the number of multi-dimensional spaces is n, and i and j represent the number of objects and attributes. The standard scaling formula for calculating an interval according to the Euclidean distance formula is:


(1) $$d\left(a_{i},a_{j}\right)=\sqrt{\sum_{r=1}^{n}{\left(a_{ir}-a_{jr}\right)}^{2}}$$

The standard scaling formula for calculating an interval based on the Manhattan distance formula is:


(2) $$d\left(a_{i},a_{j}\right)=\sum_{r=1}^{n}\left|a_{ir}-a_{jr}\right|$$

In the Euclidean n-dimensional space model, the cosine variable can also be used as a way to calculate the standard scale of the interval. The calculation formula is:


(3) $${\text{O}}_{\cos}\left(a_{i},a_{j}\right)=\frac{\sum_{r=1}^{n}\left(a_{ir}\cdot a_{jr}\right)}{\sqrt{\sum_{r=1}^{n}a_{ir}^{2}\sum_{r=1}^{n}a_{jr}^{2}}}$$

Murkowski distance is a generalization of the Manhattan distance formula, and its calculation formula is:


(4) $$d\left(a_{i},a_{j}\right)=\sqrt[{x}]{\sum_{r=1}^{n}{\left(a_{ir}-a_{jr}\right)}^{x}}$$

Non-linear data, as distinguished from linear data, use scaled variables. The principle of the scale scaling metric: It assumes a dataset A of length L. φ is the number of the element attribute. For any two elements a and b in this dataset, there is a dissimilarity relationship:


(5) $$d\left(a_{i},a_{j}\right)jr=\sum_{r=1}^{n}\frac{\left|a_{ir}-a\right|}{\left|a_{ir}+a_{jr}\right|}$$

Non-linear data, as distinguished from linear data, use scaled variables. The principle of the scale scaling metric: It assumes a dataset A of length L. For any two elements a and b in this dataset, there is a dissimilarity relationship:


(6) $$d\left(a,b\right)=\frac{\sum_{n=1}^{L}\sigma_{ab}{}^{\varphi }d_{ab}{}^{\varphi }}{\sum_{n=1}^{L}\sigma_{ab}{}^{\varphi }}$$

In reality, the data that needs to be processed is not only from a linear perspective and a non-linear perspective, but also multivariate data. Therefore, it uses binary variables for calculations on such data. Binary variables are divided into two states: 0 and 1.0 means the attribute will not appear, 1 means the attribute will appear.

It assumes that in the n-dimensional space, A represents the number of attributes of ^*a*_ir_=*a*_jr_=1^, B represents the number of attributes of ^*a*_ir_=1,*a*_jr_=0^, C represents the number of attributes of ^*a*_ir_=0,*a*_jr_=1^, and D represents the number of attributes of ^*a*_ir_=*a*_jr_=1^. For data with symmetric attributes, the calculation formula for evaluating the dissimilarity of two sample data is:


(7) $$E\left(a_{i},a_{j}\right)=\frac{A+B}{A+B+C+D}$$

For data with asymmetric attributes, the calculation formula for evaluating the dissimilarity of two sample data is:


(8) $$E\left(a_{i},a_{j}\right)=\frac{A}{A+B+C}$$

On the basis of binary variables, in order to deal with some data sets with slightly more complex data, nominal variables are derived. It assumes that the number of data with nominal attributes is F, e is the total number of attributes of an element under the attribute, and f is the number of elements matched. Then the dissimilarity formula between any two elements in the attribute dataset is:


(9) $$d\left(a_{i},a_{j}\right)=\frac{e-f}{e}$$

The formula for calculating the similarity between any two elements in the attribute dataset is:


(10) $$S\left(a_{i},a_{j}\right)=1-d\left(a_{i},a_{j}\right)$$

According to the characteristics of the data processed by the clustering algorithm, before clustering the data, it is necessary to standardize the data in the data. The calculation formula according to the measurement method is:


(11) $$D_{i\varphi }=\frac{a_{i\varphi }-n_{\varphi }}{q_{\varphi }}$$

*q*_φ_ means the mean absolute deviation, *n*_φ_ means the mean, m represents the number of attributes, and the formula for calculating the mean absolute deviation is:


(12) $$q_{\varphi }=\frac{\left|a_{1\varphi }-n_{\varphi }\right|+\left|a_{2\varphi }-n_{\varphi }\right|+\ldots \left|a_{m\varphi }-n_{\varphi }\right|}{m}$$

The formula for calculating the mean is:


(13) $$n_{\varphi }=\frac{a_{1\varphi }+a_{2\varphi }+\ldots a_{m\varphi }}{m}$$

The standard metric value calculation formula is:


(14) $$\lambda_{i\varphi }=\frac{a_{i\varphi }-n_{\varphi }}{q_{\varphi }}$$

After the preliminary work of clustering is ready, since clustering is to associate data with similarities together, in the process of data iteration, a criterion function needs to be used to determine whether to stop the iterative process or not. It assumes that the distance between any element a in a certain cluster *W*_*i*_ after the data set is classified and the cluster center *w*_*i*_ is represented as *d*(*a*, *w*_*i*_). The formula of the error sum of squares criterion function is:


(15) $$P_{W}=\sum_{i=1}^{r}\sum d{\left(a,w_{i}\right)}^{2}$$

(16) $$w_{i}=\frac{\sum_{m=1}^{m_{i}}a_{j}}{m_{i}}$$

When the sum of squared errors is larger, it indicates that the objects in the cluster are more deviated from the center, and the clustering effect is not obvious. When the error sum of squares is smaller, the objects in the cluster are more compact, and the clustering effect is better.

It assumes that κ_*j*_ represents the average squared distance between elements within a cluster. Then according to the weighted average squared distance and the criterion, the function formula is:


(17) $$\varepsilon_{i}=\sum_{i=1}^{r}a_{i}\cdot \kappa_{j}$$

The formula for calculating the average squared distance between elements in a cluster is:


(18) $$\kappa_{j}=\frac{2\left(\sum a\in w_{i}\sum a^{\prime }\in w_{j}|a-a^{\prime }{|}^{2}\right)}{m_{j}\left(m_{j}-1\right)}$$

The inter-class data in the clustered data is calculated using the inter-class distance and criterion. In this method, two quantities are used, the inter-class distance criterion κ_*a_1_*_ and the weighted inter-class distance criterion κ_*a_2_*_. The calculation formula is:


(19) $$\kappa_{a_{1}}=\sum_{j=1}^{r}{\left(\mu_{j}-\mu \right)}^{T}\left(\mu_{j}-\mu \right)$$

(20) $$\kappa_{a_{2}}=\sum_{j=1}^{r}p{\left(\mu_{j}-\mu \right)}^{T}\left(\mu_{j}-\mu \right)$$

p is the prior probability, μ is the mean vector of the whole data.

### Canopy clustering algorithm

**FIGURE 9:**
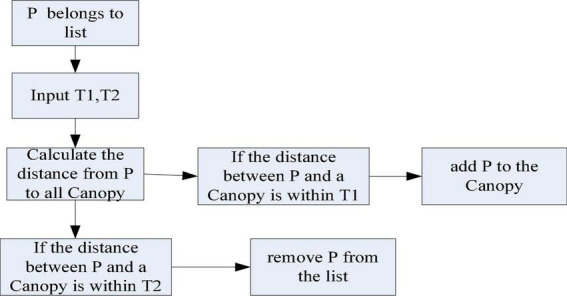
Canopy algorithm flow.

The advantage of the Canopy clustering algorithm is that it does not need to specify the value of k (the number of clusters) and can be performed in multiple threads. Therefore, its clustering speed is faster, but the accuracy is lower (Xia et al., 2020). It processes the data using the Canopy clustering algorithm to group similar objects into a subset. This subset is called Canopy. The Canopy algorithm flow is shown in Figure 9.

Table 2 shows the classification of clustering algorithms and the principles and representative algorithms of each classification algorithm. From the classification in the table, the basic principle of cluster analysis is to divide the data. It progressively improves the clustering quality and approaches the local optimal solution (Atashi et al., 2017). According to the characteristics that need to be dealt with in reality, there are also certain requirements for clustering. Its specific requirements are shown in Table 3.

According to the algorithm flow of Canopy in Figure 9, when using the Canopy algorithm for data analysis, it is first necessary to select T1 and T2 to calculate the distance between a certain sample and all Canopy subsets. It classifies samples with distances within T1 into this Canopy subset. It removes samples whose samples are within T2 from the dataset. It keeps doing these two steps until the dataset is empty and the clustering is complete.

**FIGURE 10:**
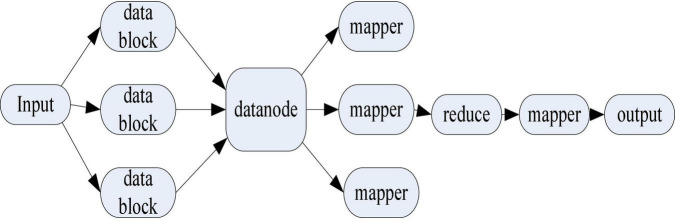
Implementation path of parallelized Canopy clustering algorithm.

The Canopy clustering algorithm was initially proposed to deal with larger-scale data. When the scale of data continues to increase, the Canopy clustering algorithm is also difficult to support. Therefore, it is necessary to use the Map Reduce software framework to optimize the Canopy clustering algorithm to adapt to large-scale data. Its realization path is shown in Figure 10.

Figure 10 shows the implementation path of the parallelized Canopy clustering algorithm. According to the structure of the graph, the parallelized Canopy clustering algorithm first inputs the data set and initially clusters it into data blocks. It distributes data blocks to datanodes, which are then processed concurrently with map. It performs the reduce operation to merge the clustering results, and then uses the map to get the final clustering results.

## Consumer psychology experiment and analysis

### Experimental design and indicators

Questionnaire survey was used in the experiment. The questionnaires were distributed in the form of random distribution on the Internet and random distribution on the ground. A total of 815 questionnaires were collected. The questionnaire design is mainly divided into the basic situation of the respondents, commodity attributes, and consumer psychology. Among them, the product attributes of the questionnaire content and the design of consumer psychology content are shown in Table 4.

**TABLE 4:** Commodity attributes and consumer psychological content indicators.

	Variable							
Product attributes	X1	X2	X3	X4	X5	X6	X7	X8
	Discount	Price	Grade	Sales	Evaluate	Distance	Brand	Unique
Consumer psychology	Y1	Y2	Y3	Y4	Y5			
	Convenience psychology	Affordable psychology	Quality psychology	Personality psychology	Worry			

Table 4 is the main content of commodity attributes and consumer psychology involved in the questionnaire. The commodity attributes are discount, price, grade, sales volume, evaluation, distance, brand, and uniqueness variable indicators. The consumer psychology is convenience, benefit, quality, individuality and worry. Convenience means that consumers take into account the reduction of time and labor costs to the greatest extent possible when consuming. Affordable means that consumers are relatively price-sensitive and pursue higher cost performance. Quality-seeking means that consumers have high psychological demands for product quality, reputation, and brand. Personality refers to consumers who are more concerned about the unique and personalized needs when choosing products. Worry refers to the lack of trust in the product or the merchant at the time of consumption.

**FIGURE 11:**
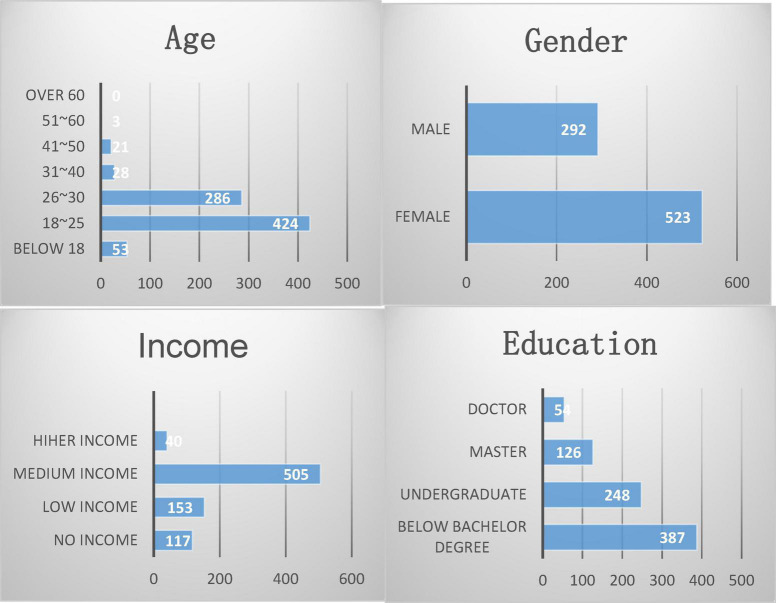
Basic information of respondents.

The basic information of the respondents is shown in Figure 11.

As can be seen from Figure 11, the sample coverage of this questionnaire survey is relatively narrow. The educational level and income of the participants in the questionnaire cannot represent the general level in China.

This experiment will analyze the consumer psychology in the digital economy based on the questionnaire survey, and use the Canopy clustering algorithm to build a consumer psychology prediction model in the digital economy.

### Data preprocessing

In real data, there are often some abnormal data. For example, in the questionnaire, the same option is selected or the option is selected regularly, and one person fills in multiple copies. During data mining, these data should be treated as outliers. When the data is transmitted in the algorithm, it is often mixed with noise, so it is necessary to eliminate the noise. This paper uses wavelet noise reduction. It makes the data localized and subdivided by scaling the translation and multi-angle transformation of the data set to achieve the effect of denoising and denoising.

According to the variables of the questionnaire, it consults relevant literature and constructs a correlation between commodity attributes and consumer psychology. The specific corresponding situation is shown in Table 5.

**TABLE 5:** Relationship between commodity attributes and consumer psychology.

Product attributes	Consumer psychology
Discount (X1)	Affordable psychology (Y2), worry (Y5)
Price (X2)	Quality psychology (Y3), affordable psychology (Y2), worry (Y5)
Grade (X3)	Quality psychology (Y3), worry (Y5)
Sales (X4)	Quality psychology (Y3), worry (Y5), personality psychology (Y4)
Evaluate (X5)	Quality psychology (Y3), worry (Y5), convenience psychology (Y1)
Distance (X6)	Convenience psychology (Y1)
Brand (X7)	Quality psychology (Y3), worry (Y5), personality psychology (Y4), convenience psychology (Y1)
Unique (X8)	Personality psychology (Y4)

From the relationship between commodity attributes and consumer psychology in Table 5, it can be seen that different consumer psychology has different attitudes toward commodity attributes.

### Design and analysis of digital economy consumer psychology prediction model based on canopy clustering algorithm

Combined with the analysis of commodity attributes and consumption psychology and the Canopy clustering algorithm, the construction process of the digital economy consumer psychology prediction model based on the Canopy clustering algorithm is as follows:

(1) It randomly selects 300 questionnaires and inputs the questionnaire data;

(2) It performs preliminary clustering according to commodity attributes and divides it into commodity attribute data blocks;

(3) It processes the data blocks concurrently with map, and obtains the consumer psychology clustering under different commodity attributes;

(4) reduce merges the clustering results;

(5) It gets the final clustering result;

(6) It calculates the probability distribution of consumer psychology.

Its prediction result evaluation index is evaluated according to the mean absolute value error index. It uses *t*_*predict*_ to represent the predicted probability and *t*_*actual*_ to represent the actual probability. The mean absolute value error formula is:


(21) $$M=\frac{\sum_{i=1}^{m}\left|t_{predict}-t_{actual}\right|}{m}$$

**FIGURE 12:**
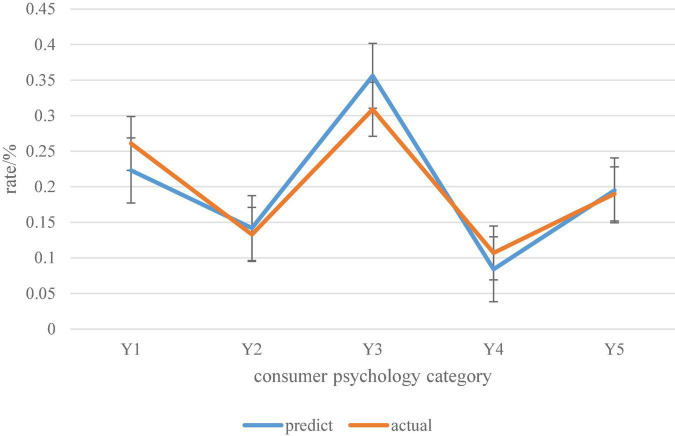
Predicted probability distribution and actual probability distribution of consumer psychology categories based on Canopy clustering algorithm.

The predicted probability distribution and actual probability distribution statistics of consumer psychology categories based on the Canopy clustering algorithm are shown in Figure 12.

It can be seen from the probability distribution curve in Figure 12 that the predicted probability distribution of consumer psychology categories based on the Canopy clustering algorithm is not much different from the actual probability distribution. Therefore, it can be considered that the digital economy consumer psychology prediction model of the Canopy clustering algorithm has certain adaptability and stability.

## Discussion

The development of digital economy is the trend of economic development. In the context of digital consumption, consumers are becoming more and more active in their behavior. The alliance between consumers can even affect the selling price and quality of digital products. The evaluation of various product attributes is more transparent. Therefore, consumer psychology evaluation under the development of digital economy is particularly important. This paper conducts a screening evaluation based on the digital consumption psychology in the context of the digital economy. It establishes the link between commodity attributes and consumer psychology. Based on the Canopy clustering algorithm, it builds a consumer psychology prediction model in the context of the digital economy, and evaluates the prediction data. For the screening of consumer psychology is not perfect, this is the lack of this paper. However, due to the lack of statistical investigation, the consumer psychology for screening is not perfect, which is the shortcoming of this paper. Compared with the traditional statistical analysis method, the clustering algorithm has stronger data analysis ability and faster analysis process. Although the Canopy clustering algorithm is simply optimized in this paper, a comparative model is not established. And due to the limitations of the questionnaire survey, the survey population does not conform to the law of sampling survey and is not representative, which is the shortcoming of this paper.

## Conclusion

This paper builds a consumer psychology prediction model under the digital economy model based on the Canopy clustering algorithm, and evaluates the results of the prediction model. The digital economy in the Internet era has developed rapidly and has become a new economic form. It is an important force for global economic development. In the context of the digital economy model, through the investigation and research on the digital economy and the review of related literature on consumer psychology in the network environment, this paper mainly summarizes the consumer psychology in the context of the digital economy as convenience, benefit, quality, individuality, worry. It clusters the data according to the Canopy clustering algorithm. According to the clustering results of consumer psychology, it calculates the predicted probability distribution of consumer psychology. It also derives the actual probability distribution based on the questionnaire results. Among them, the predicted result of convenience psychology is 0.223, and the actual result is 0.261. The predicted result of Affordable Psychology was 0.142, and the actual result was 0.133. The predicted result of qualitative psychology is 0.356, and the actual result is 0.309. The predicted result of personality psychology is 0.084, and the actual result is 0.107. The predicted result of worry psychology is 0.195, and the actual result is 0.19. Finally, it is concluded that the digital economy consumer psychology prediction model based on Canopy clustering algorithm has certain applicability.

## Data availability statement

The original contributions presented in this study are included in the article/supplementary material, further inquiries can be directed to the corresponding author.

## Ethics statement

Ethical review and approval was not required for the study on human participants in accordance with the local legislation and institutional requirements. Written informed consent from the patients/participants was not required to participate in this study in accordance with the national legislation and the institutional requirements.

## Author contributions

YZ carefully designed and wrote the manuscript. PR design questionnaire and collect data, and conduct preliminary statistical analysis, as well as model screening. JZ and YZ carried out statistical analysis of data, model determination, and experiment. JZ assists in writing articles. All authors gave final approval of the version to be published and agreed to be accountable for all aspects of the work.
